# Low cost, microcontroller based heating device for multi-wavelength differential scanning fluorimetry

**DOI:** 10.1038/s41598-018-19702-6

**Published:** 2018-01-23

**Authors:** Jo Hoeser, Emmanuel Gnandt, Thorsten Friedrich

**Affiliations:** 1 Institut für Biochemie, Albert-Ludwigs-Universität Freiburg, Albertstr. 21, 79104 Freiburg i. Br., Germany; 2 Present Address: Luxembourg Science Center, 50 rue Emile Mark, 4620 Differdange, Luxembourg

## Abstract

Differential scanning fluorimetry is a popular method to estimate the stability of a protein in distinct buffer conditions by determining its ‘melting point’. The method requires a temperature controlled fluorescence spectrometer or a RT-PCR machine. Here, we introduce a low-budget version of a microcontroller based heating device implemented into a 96-well plate reader that is connected to a standard fluorescence spectrometer. We demonstrate its potential to determine the ‘melting point’ of soluble and membranous proteins at various buffer conditions.

## Introduction

Thermal Shift Assays were developed as a high-throughput screening method for drug discovery^1–3^. The ligand, commonly a small molecule, interacts with the protein inducing ligand-protein interaction based conformational stabilization. Ligand-binding and protein stability are energetically coupled and are monitored in multiple ways^1–3^. Thermal shift assays are performed using differential scanning calorimeters (DSC), following the change in heat capacity induced by thermal denaturation^4^. Alternatively, heatable fluorescence spectrometers or RT-PCR machines are used to detect conformational changes upon thermal protein unfolding and denaturation. For differential scanning fluorimetry (DSF) an extrinsically fluorescent probe has to be added to the sample^5^.

The unfolding of soluble proteins is monitored by the interactions of its hydrophobic core with the hydrophobic probe SYPRO Orange, initially developed as high-sensitivity PAGE stain^6,7^. However, it cannot be used to monitor the unfolding of membrane proteins due to its binding to the protein-associated detergent. Instead, 7-Diethylamino-3-(4′-Maleimidylphenyl)-4-Methylcoumarin (CPM) is used as a thiol-reactive probe that was initially developed as a stain for tissue samples^7^. Upon unfolding, an increasing number of thiols are exposed and react with the probe^8^. To monitor protein aggregation rather than unfolding, the ProteoStat dye is on the market^9^. Furthermore, the denaturation of special proteins are monitored by the release of fluorescent cofactors, such as flavins as employed in the ThermoFAD assay^10^. Currently available RT-PCR machines use matching LED-phototransistor pairs to monitor fluorescence changes allowing the measurement of e.g. SYPRO Orange fluorescence. Other probes such as CPM cannot be detected with the given excitation/emission wavelength pairs. On the contrary, most fluorescence spectrometers with a variable setting of the wavelength are limited in the maximal heating temperature, strongly limiting the use of such devices for DSF experiments.

Here, we introduce a microcontroller driven heating device that is incorporated into the plate reader of a commercial fluorescence spectrometer. The heater is designed to fit between plate-holder and plate and we developed software to control the spectrometer and the heater in parallel, to fully automate the measurement process. Alternatively, using a simplified software version, it can be used as a stand-alone heater and incubator for plates.

## Materials and Methods

### Heating device and software

A commercially available fluorescence spectrometer (LS-55, Perkin-Elmer) equipped with a 96-well plate reader accessory was used in all experiments. To assure an accurate heat regulation, a negative temperature coefficient (NTC) glass-bead thermistor was glued between a silicone heat pad and a thin sheet of stainless steel using Kapton tape. The heat pad was operated using a solid-state relay and the Arduino UNO R3 open-source prototyping platform was used as microcontroller. The heat pad was tightly inserted into the bottom of the 96-well plate (OptiPlate-96, Perkin Elmer). The entire setup was then inserted into the plate reader using a 3D-printed holder (Supplementary Figs 1 and 2 and Supplementary Table 1). Care was taken that the setup is easily removable and no modifications to the fluorescence spectrometer were needed. The microcontroller was programmed to continuously measure the temperature of the heat pad and to adjust the heating power by means of the proportional-integral-derivative (PID) approach. To minimize signal noise, several data points were used to calculate the average of the actual temperature during the measurement. This value is transmitted to a PC *via* serial connection and evaluated by additional software. *Vice versa*, a new target temperature is transmitted to the microcontroller *via* serial connection at any time (Supplementary Fig. 2, bottom left). To achieve a fully automated setup for the differential scanning fluorimetry assay, software controlling the heating device as well as the fluorescence spectrometer *via* PC using two different serial ports was used. The software controlled cycling through a routine composed of heating, incubation and measurement to a maximal temperature of 95 °C. Incubation and measurement of each temperature step takes 5 min and one complete measurement from room temperature to 95 °C using 3 °C steps takes approximately 2 h. All data were saved into a single output file (Microsoft Excel file-type) using one sheet per temperature step (Supplementary Fig. 2, top left).

We also wrote a simplified software to control the heating device, regardless of the spectrometer used. Here, the user only needs to specify the temperature steps and the maximal temperature, while the software continuously plots the actual heat pad temperature and shows the incubation time. With this setup, it is possible to heat up and incubate any 96-well plate reproducibly. In that case, the respective software of the spectrometer is needed to measure the fluorescence (i.e. Perkin-Elmer FLWinLab). Full access to hardware (schematics, part lists, and constructional drawings), software (Arduino firmware, control software and source codes) and possible future updates is provided through https://github.com/JoHoeser/SIMPLE-DSF.

### Temperature validation

In order to verify the β-value of the NTC and the temperature characteristics of the heat pad, its surface temperature at the centre and each of the corners was determined during the heating process using an IR thermometer (Etekcity Lasergrip 1080). This calibration was used to correct the heating parameters in the Arduino firmware. The sample temperatures at different positions in the used 96-well plates were evaluated using a digital temperature logger (testo 176 T4) and four type K NiCr-Ni temperature probes (testo 0602.5792). The well temperature readings were averaged and plotted against the temperature set in the software.

### Sample preparation

Depending on the assay, the reaction mixture contained 1–100× SYPRO Orange (SYPRO Orange Protein Gel Stain, Sigma-Aldrich) and 10 nM-10 µM protein mixed in the desired buffer. The optimal amounts of SYPRO Orange and protein were determined in a two-dimensional concentration grid. The reaction mixture without protein was used as blank. All samples were prepared in triplicates on ice, overlaid with silicone oil (M-350, ROTH) to prevent evaporation and measured at excitation and emission wavelengths of 467 ± 3 and 570 ± 3 nm, respectively, with a 530 nm emission filter. The same measurements were performed with a RT-PCR machine (CFX96 Touch, BioRad) using a 96-well plate (Hard-Shell PCR Plates 96-well, BioRad) sealed with a clear adhesive foil (Microseal ‘B’ Adhesive Seals, BioRad). The SYBR/FAM filter (470 ± 20 nm excitation, 520 ± 10 nm emission) setting was used. The plate was incubated at 15 °C for 3 min and the temperature increased in 1 °C steps followed by 30 s incubation prior to fluorescence determination.

Alternatively, the reaction mixture comprised 10 µM CPM (life technologies) and protein containing a total of 2 µM cysteine residues in the desired buffer. The reaction mixture without protein was used as blank. All samples were treated as described above. Excitation and emission wavelength of 384 ± 3 and 470 ± 3 nm, respectively, were used in combination with a 430 nm emission filter. Detergent screening using cytochrome *bd-I* from *E. coli* was carried out by adding the tenfold critical micellar concentration (CMC) of each detergent to the samples (JBScreen Detergents #CD-103, Jena Bioscience). Sample plates for anoxic measurements were entirely prepared in an anoxic tent (Coy).

The ThermoFAD method was carried out as described using a RT-PCR machine^10^. The measurement was repeated using the microcontroller based device using excitation and emission wavelength of 470 ± 10 and 530 ± 3 nm, respectively, without emission filter.

### Data evaluation and melting point determination

Datasets recorded under a distinct condition were averaged and plotted using Origin 2018 (Originlab). The melting temperature of each sample was determined by applying the built-in ‘Boltzmann’ fit to the curve. In case of two inflection points the ‘DoubleBoltzmann’ fit was used. Due to decreasing fluorescence after full denaturation of the sample, these data were not used for curve fitting to improve fit quality. In rare cases, where the fits did not converge, the 2^nd^ derivative was plotted and the zero-crossing was used as melting point.

## Results

### Temperature validation

**Figure 1:**
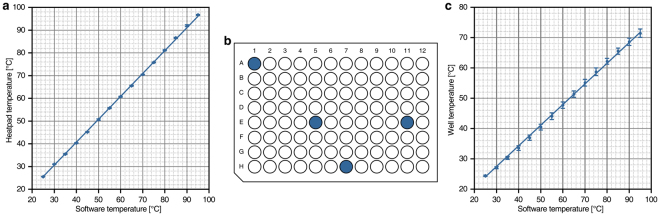
Validation of heater and well temperatures. **(a)** Temperature profile of heat pad surface after calibration. **(b)** Sample wells that were used for temperature monitoring validation. **(c)** Temperature profile inside sample wells shown in **b** during DSF experiment.

Temperature validation revealed a strictly linear correlation between the set temperature (x) and the average heat pad temperature (y) at different positions on the plate. The relation obtained was very close to the desired y = x (y = 1.008 ± 0.004·x + 0.366 ± 0.225). With an average standard deviation of 0.2 °C, the heat pad temperature can be considered sufficiently accurate for DSF assays (Fig. 1a).

The temperature inside the sample wells was analysed using multiple temperature probes on positions in the centre, the corner and on both sides of the plate (Fig. 1b). Again, a linear correlation between the set temperature and the well temperature (y = 0.681 ± 0.007·x + 7.034 ± 0.271) was obtained. Although the maximal well temperature (71.5 °C) is significantly lower than the heat pad temperature (95 °C), the samples were continuously heated in a reproducible manner (Fig. 1c). An average standard deviation of 0.8 °C among all wells was recorded, which is only slightly higher than that of the heat pad temperature. Additionally, the experimentally determined slope enables the calculation of the actual well temperature.

### Comparison with commercially available heater devices

**Figure 2:**
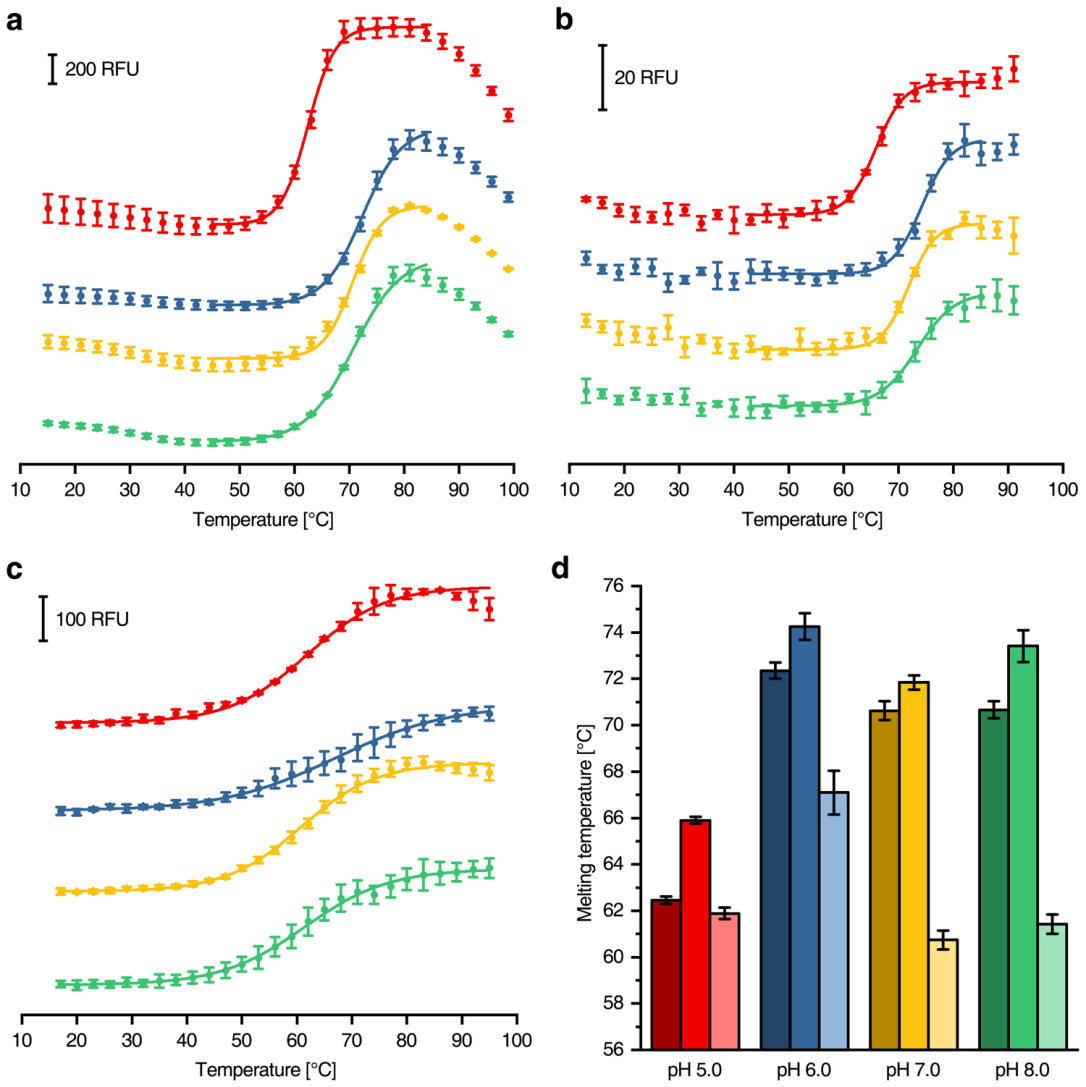
Differential scanning fluorimetry of β-amylase using different dyes and devices. **(a)** Melting curves obtained using 1 µM β-amylase with 10× SYPRO Orange in combination with a RT-PCR machine (data points recorded every 1 °C, but 3 °C steps shown for illustration purposes). (**b**) The same measurement as in **a** using the microcontroller based device. (**c**) CPM-fluorescence of 1 µM β-amylase measured with the microcontroller based device. (**d**) Comparison of the melting temperatures obtained from (**a**) (dark colours), (**b**) (medium colours) and **c** (light colours). The buffers were set to pH 5.0 (red), pH 6.0 (blue), pH 7.0 (yellow) and pH 8.0 (green).

Melting curves of β-amylase from *Ipomoea batatas* (Sigma) in various buffers recorded on a RT-PCR machine using the SYPRO Orange fluorescence (Fig. 2a) were compared to those from our microcontroller based device (Fig. 2b). Similar shaped melting curves and standard deviations were achieved with both devices (Fig. 2d). The thermal stability decreased from pH 6.0 to pH 7.0–8.0, however, it was clearly reduced at pH 5.0. Generally, the melting temperatures determined by the microcontroller based device were slightly increased compared to those recorded with the RT-PCR machine. This might be caused by the bottom shape of the 96-well plates, hampering heat transfer. Nevertheless, the melting temperatures obtained with both machines were highly similar, confirming the function and accuracy of the microcontroller based device.

Furthermore, the thermal stability of β-amylase with CPM as fluorescent probe was analysed. This measurement cannot be performed with RT-PCR machines, since they are lacking the corresponding excitation- and emission wavelength pairs. Again, distinct melting curves were obtained (Fig. 2c), although they are less steep than those determined by using SYPRO Orange (Fig. 2a,b). This is most likely caused by the stepwise reaction of cysteine residues, which is not resolved leading to a global melting temperature, in contrast to the more concerted exposure of the hydrophobic protein core. Nevertheless, the melting temperature depended on the pH in the same way as obtained with SYPRO Orange as dye. The highest thermal stability was obtained at pH 6.0 but with this probe, high pH values had a more severe impact on thermal stability demonstrating the differences in the detection method (Fig. 2d). In summary, the observed melting temperatures were similar by using the different fluorescent dyes and were in good accordance to the activity maximum of β-amylase at pH 6.2^11^.

### Application to membrane proteins

**Figure 3:**
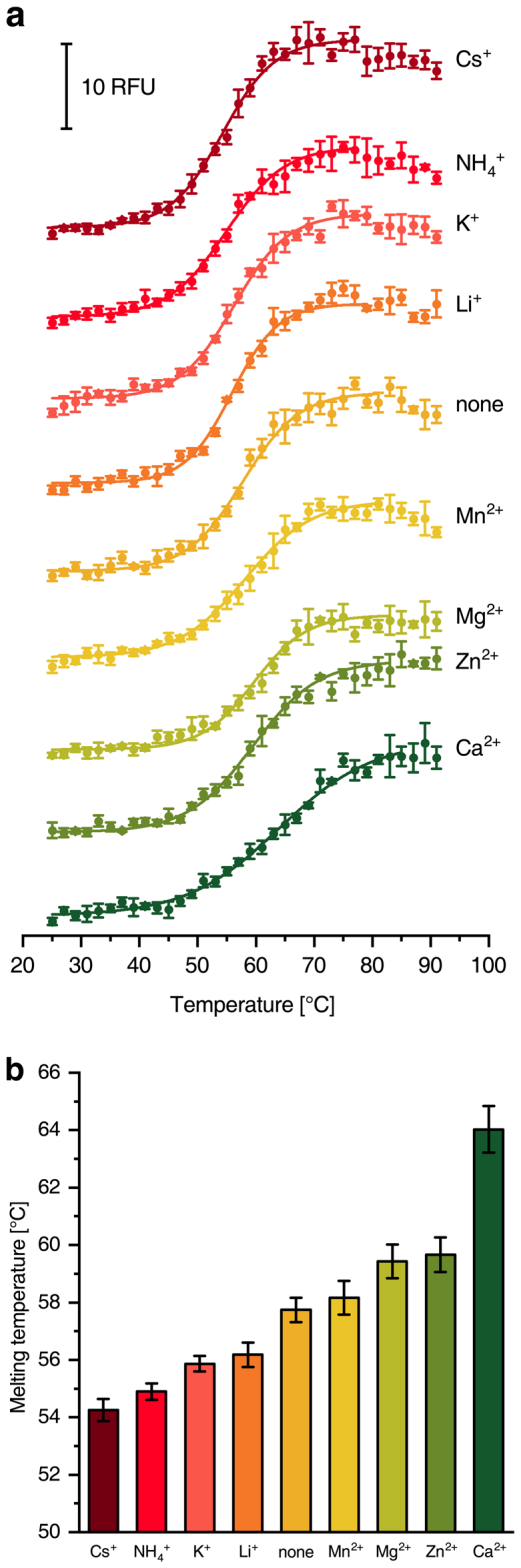
Melting curves of *E. coli* complex I with different salt additives. **(a)** CPM-fluorescence of 110 nM complex I in the presence of various salts. 5 mM of mono- and divalent cation chlorides were added to the standard buffer^16^. (**b**) Shows the respective melting temperatures obtained from (**a**).

To challenge the capabilities of the microcontroller based device the denaturation of *Escherichia coli* respiratory complex I, a multi-subunit 535 kDa membrane protein with two [2Fe-2S], seven [4Fe-4S] clusters and a FMN cofactor^12–15^, was analysed using CPM as fluorescent probe. The destabilization of the complex by monovalent cations and its stabilisation by divalent cations was determined (Fig. 3). It turned out that Ca^2+^ exerted a strong stabilisation on the complex while Mg^2+^, Zn^2+^ and Mn^2+^ showed a small stabilising effect. These data are consistent with the literature, with Mg^2+^ being known as salt additive for protein purification and Ca^2+^ providing a more stable preparation of bacterial complex I^14,16,17^. Zn^2+^ is known to inhibit the proton transfer pathways of complex I, thereby compacting the structure of the complex^12^.

**Figure 4:**
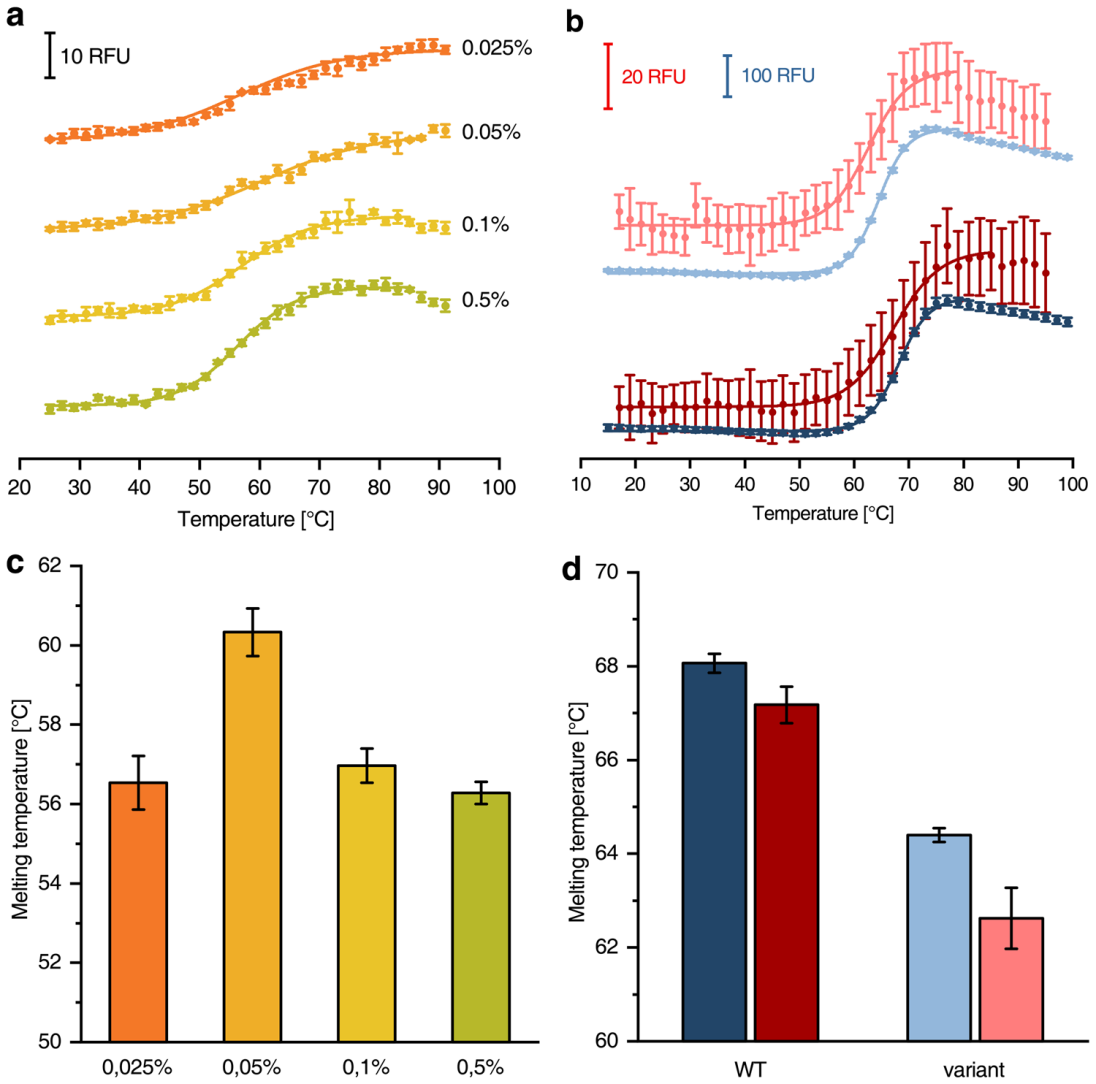
Melting curves of *E. coli* complex I at different detergent concentrations and ThermoFAD melting curves of the wild-type enzyme and a variant. **(a)** CPM-fluorescence of 110 nM complex I in the presence of various DDM concentrations (ascending from orange to green). (**b**) ThermoFAD^10^ melting curves using 750 nM wild-type complex I (dark colours) or an equal amount of the V96P/N142M variant^14^ (light colours). The measurement was carried out with a RT-PCR machine (blue, data points recorded every 1 °C, but 2 °C steps shown for illustration purposes) and the microcontroller based device (red). (**c** and **d**) Show the melting temperatures obtained from (**a**) and (**b**), respectively.

The optimal concentration of the detergent *n*-Dodecyl β-d-maltoside (DDM) for complex I purification was determined using CPM as fluorescent probe (Fig. 4a,c). The highest protein stability was found for 0.05% DDM, representing 5.7-fold CMC (0.0087%)^12^.

### Comparison with the ThermoFAD assay

In addition, the release of the fluorescent FMN cofactor from *E. coli* respiratory complex I upon heating was measured and compared to the melting temperature of a complex I variant with mutations nearby the flavin binding site by means of the ThermoFAD assay^10^, using the RT-PCR device and the microcontroller based device (Fig. 4b,d). The progression of the melting curves were similar, however, the microcontroller based measurement showed a five times lower fluorescence intensity caused by the setup of the fluorescence spectrometer. Nevertheless, the melting temperatures determined with both methods demonstrated the effect of the mutations on the destabilization of binding the flavin cofactor. These data obtained are consistent with published results^15^.

### Detergent screening for membrane proteins

**Figure 5:**
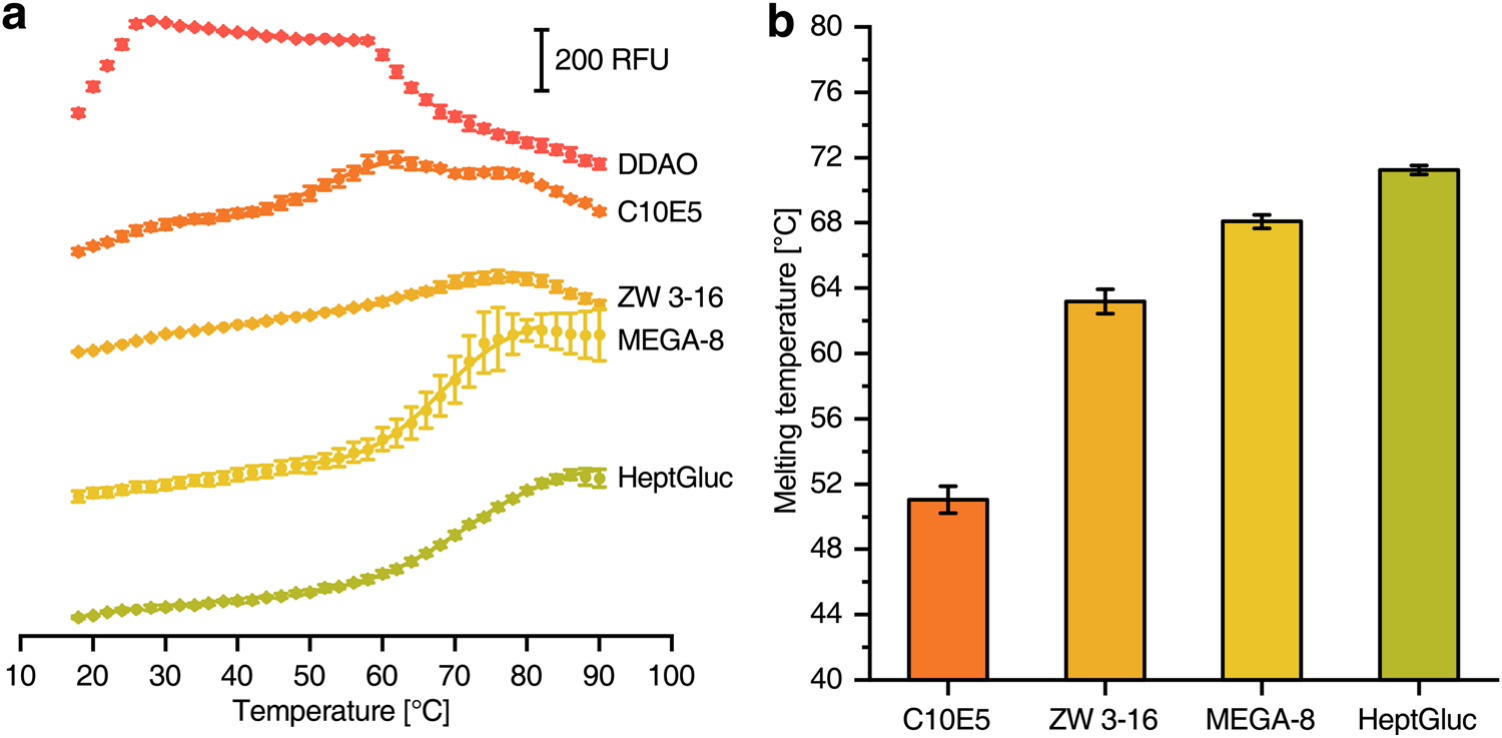
Melting curves of *E. coli* cytochrome *bd* oxidase in the presence of different detergents. **(a)** CPM-fluorescence of 285 nM cytochrome *bd* oxidase in buffers containing different detergents. **(b)** Shows the respective melting temperatures obtained from (**a**). No melting temperature could be determined for DDAO. DDAO: *n*-Decyl-N,N-Dimethylamine-N-Oxide; C10E5: Pentaethylene Glycol Monodecyl Ether; ZW 3–16: Zwittergent 3–16; MEGA-8: Octanoyl-N-Methylglucamide, HeptGluc: *n*-Heptyl-β-d-glucopyranoside.

CPM based melting curves were also used to determine the optimal detergent for membrane protein preparation. The influence of different types of detergents on the *E. coli* cytochrome *bd-I* oxidase was investigated (Fig. 5). This 105 kDa integral membrane protein consists of three subunits and contains three heme groups as cofactors^18^. The preparation of the enzyme complex was most stable in sugar-based detergents (i.e. MEGA-8, heptylglucoside). Expectedly, more de-lipidating detergents such as polyethers (i.e. C10E5) or ionic ones (i.e. Zwittergent 3–16) generally resulted in poor thermal stability. Interestingly, it was not possible to obtain reasonable melting curves using amineoxides (i.e. DDAO), which is most likely caused by an interaction of the positively charged detergent with the fluorescent probe that was also clearly detectable in the blank samples.

### Optimisation of buffer compositions for protein purification

**Figure 6:**
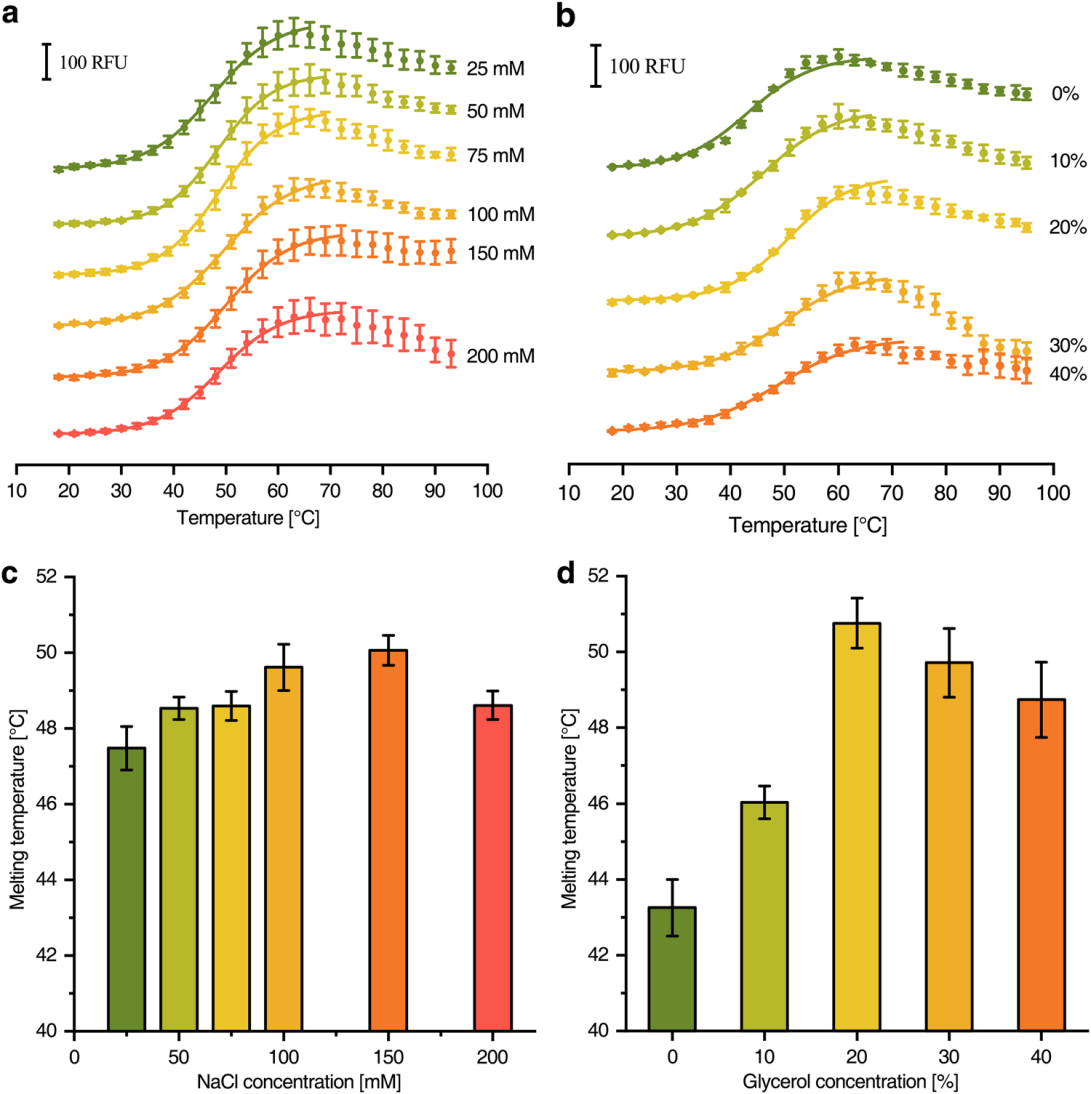
Melting curves of *D. melanogaster* cryptochrome at different buffer conditions. **(a)** CPM-fluorescence of 180 nM cryptochrome in buffers with various NaCl concentrations (ascending from green to orange). **(b)** CPM-fluorescence of 180 nM cryptochrome in buffers with various glycerol concentrations (ascending from green to orange). (**c** and **d**) Show the melting temperatures obtained from (**a**) and (**b**), respectively.

**Figure 7:**
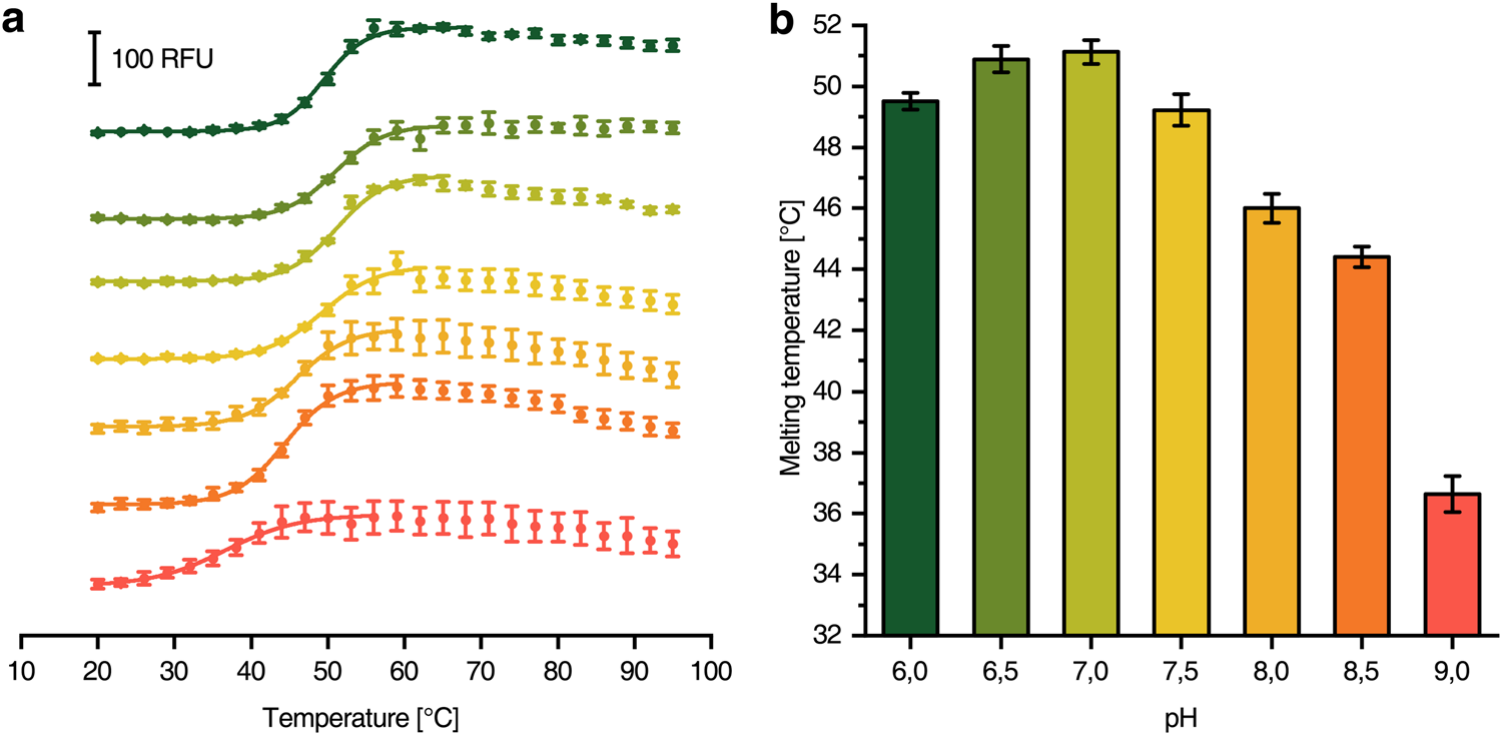
Melting curves of *A. vinelandii* cysteine desulfurase at different pH values. **(a)** CPM-fluorescence of 665 nM cysteine desulfurase in buffers with different pH values (ascending from green to red). **(b)** Shows the respective melting temperatures obtained from (**a**).

Cryptochrome from *Drosophila melanogaster*^19^ was used as example to demonstrate that the microcontroller based device is capable to screen for buffer additives that might stabilise a protein preparation. The concentration of NaCl was varied from 25 to 200 mM and that of glycerol from 0 to 40% (Fig. 6). It turned out that 100–150 mM NaCl (Fig. 6a,c) and 20% glycerol (Fig. 6b,d) yielded the highest melting temperatures. Furthermore, the lack of glycerol had a much stronger effect on the enzyme stability than the lack of NaCl. The optimal buffer pH was exemplarily screened by determining the stability of a cysteine desulfurase from *Azotobacter vinelandii* (Fig. 7)^20^. According to the melting temperatures, neutral pH values are strongly favoured for highest enzyme stability.

### Stability of proteins under anoxic conditions

**Figure 8:**
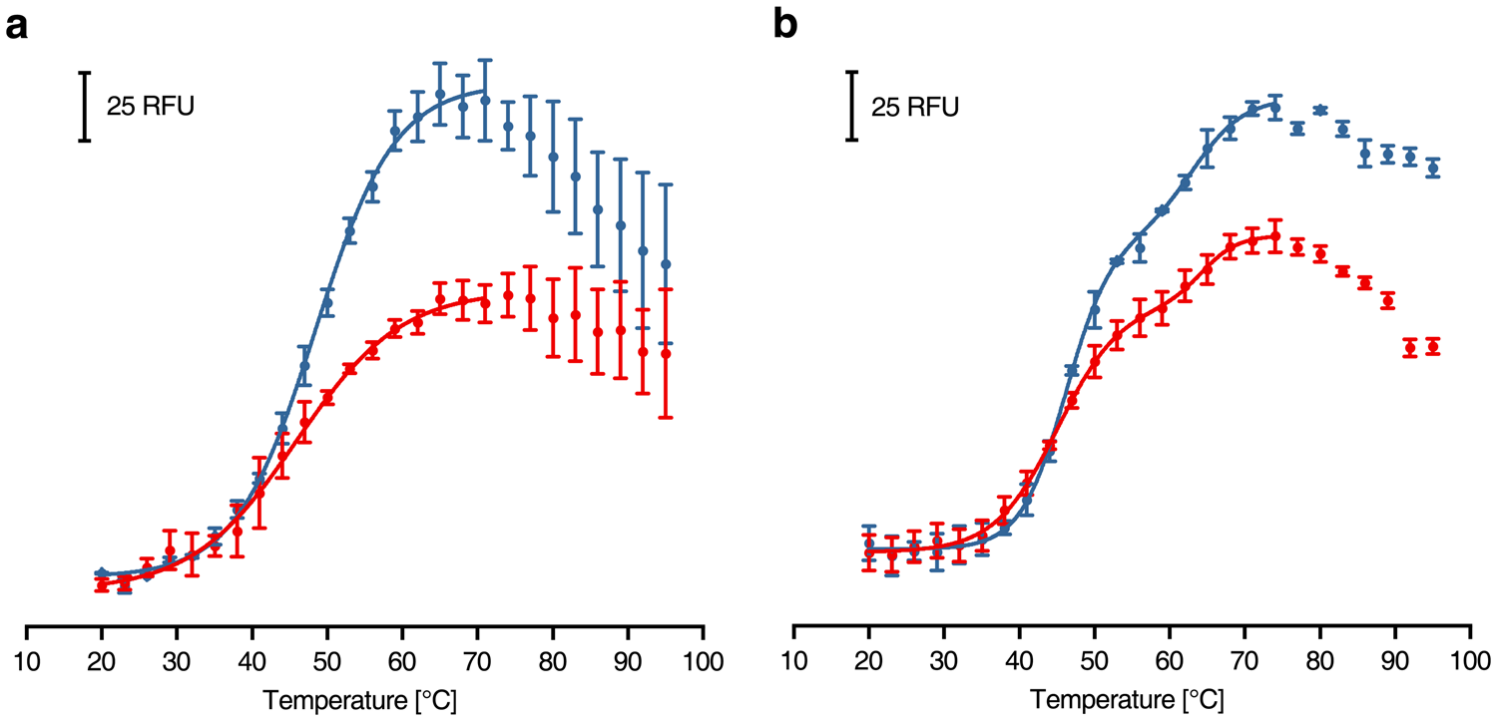
Melting curves of enzymes with oxygen sensitive cofactors under anoxic conditions (blue) and after exposure to air for 5 min (red). **(a)** CPM-fluorescence of 500 nM *A. vinelandii* FeSII. **(b)** CPM-fluorescence of 115 nM *A. vinelandii* NifDK.

The small [2Fe-2S] ferredoxin FeSII (also called Shethna protein II) from *Azotobacter vinelandii* is an oxygen sensor that undergoes a conformational change upon oxygen exposure to protect nitrogenase^21^. NifDK is the central part of the *A. vinelandii* nitrogenase containing metal clusters that rapidly decay under oxygen exposure^21^. The CPM assay was applied to these two enzymes prepared under anoxic conditions. The reduced proteins were placed in the 96-well plates in a tent under anoxic conditions, overlaid with oil and used for measurements in regular atmosphere. An aliquot of the preparations was exposed to air, centrifuged at 14.000 g for one min and placed into the wells of the plate in order to determine differences in thermal stability due to air exposure (Fig. 8). In the case of FeSII, one melting point was determined at each condition (T_m,anoxic_ = 48.3 ± 1.1 °C, T_m,oxic_ = 46.0 ± 0.5 °C), that minor change could reflect the structural change of the protein upon air exposition. Unexpectedly, two distinct melting points were obtained with NifDK (T_m1,anoxic_ = 46.1 ± 0.2 °C, T_m1,oxic_ = 45.1 ± 0.3 °C, T_m2,anoxic_ = 63.1 ± 0.5 °C, T_m2,oxic_ = 64.3 ± 0.6 °C). This might be caused by a sequential thermal denaturation of the two individual subunits possibly reflecting a significant difference in their stability. Interestingly, the melting curves of both enzymes were only slightly altered upon air exposure, while the maximal fluorescence was lowered by approximately 30%. This is most likely caused by partial denaturation of the protein upon air exposition. Aggregated protein was removed by centrifugation leaving less intact protein in the assay that in turn led to a reduced maximum fluorescence. Thus, denaturation of FeSII and NifDK due to air exposure was detectable in the difference of maximum fluorescence.

## Discussion

Using the PID based setup, a precise heat regulation of the plate reader of a commercial fluorescence spectrometer was achieved. All calibration runs stayed within a standard deviation range of well below 1 °C (Fig. 1a). Although the temperature in the sample wells is lower than the heat pad temperature due to poor thermal conductivity of the plates, the heat pad temperature was used as reference for all experiments. As a linear correlation between heat pad and well temperature was determined (Fig. 1c), it is still possible to calculate the ‘real’ melting point of a sample. However, most applications are run to determine differences between defined conditions, thus, rendering the determination of the ‘real’ melting temperature unnecessary. Furthermore, the determined melting temperature also depends on various other intrinsic factors such as sample volume and protein concentration and is therefore slightly different for each individual measurement. Thus, for comparative runs, the heat pad temperature can be reliably used for plotting melting curves.

SYPRO Orange was tested as fluorescence probe for the validation of β-Amylase thermal stability in a commercially available RT-PCR machine in comparison to our microcontroller based device (Fig. 2). We found similar results when using both machines. However, the assay with CPM as fluorescence probe was only possible in our accessory device and proved to be highly versatile. The lower apparent melting temperature obtained with our device running the CPM assay can be explained by an early proportionate unfolding of β-amylase paired with a less pronounced progression of the melting curve. The optimal pH value determined was in accordance with published results of β-amylase in all different experiments^11^.

Measurements with *E. coli* respiratory complex I were carried out for optimal cationic salt additives (Fig. 3) and detergent concentrations (Fig. 4a,c). The published destabilising effect of monovalent cations and stabilising effect of divalent cations were reflected and quantified in the experimental data^14,16,17^. It turned out that *E. coli* complex I showed the highest thermal stability at 0.05% DDM. This suggests a benefit from reducing the amount of detergent in the purification protocol of complex I in contrast to the published protocols^13,16^.

Using our microcontroller based device the ThermoFAD assay was successfully applied to complex I and a protein variant with mutations nearby the FMN binding site (Fig. 4b,d). The melting temperatures determined with a commercially available RT-PCR machine and our microcontroller based device were in good agreement with each other and with previously published results^15^.

Different NaCl and glycerol concentrations were screened for an optimal preparation of cryptochrome from the eukaryote *D. melanogaster* (Fig. 6). The determined optimal concentrations (150 mM NaCl, 20% glycerol) were in very good agreement with published purification protocols (100 mM NaCl, 20% glycerol)^19^. Furthermore, the optimal buffer pH for the preparation of the cysteine desulfurase from *A. vinelandii* was determined to pH 7.0 (Fig. 7) that only slightly differs from the published value of pH 7.4 used in the preparation^20^. In an expansion of the application, the method can also be used for optimization of detergent type and concentration as shown for respiratory complex I (Fig. 4) and cytochrome *bd-I* oxidase (Fig. 5) from *E. coli*. Throughout all the assays, the experimentally determined optimal values for protein stability were in most cases very close or identical to those experimental conditions used for protein purification demonstrating the reliability of the system. The knowledge about essential buffer optimisations are indispensable for high protein stability during protein preparation and further characterization (i.e. activity assays, ligand binding assays, crystallization, etc.) and can be realized in less than one day using our setup.

With our system the oxygen tolerance of enzymes is testable. FeSII and NifDK from *A. vinelandii*, both containing oxygen sensitive cofactors, were screened (Fig. 8). After a short exposure to air the maximal fluorescence decreased by approximately 30% with nearly unaltered melting curve shapes. It is likely that after air exposure, a part of the enzyme population aggregated and was removed by a centrifugation step in the sample preparation process. The unaltered enzyme was thermally unfolded resulting in similar denaturation curves as the anoxic samples. Here, not only the inflection points, but also the intensity of the fluorescence plateau is an essential parameter containing information.

By placing the low-cost heater element into a fluorescence spectrometer reproducible melting curves with the precision of a commercial RT-PCR device were obtained. Our setup is not limited in wavelength selection enabling the determination of melting curves using non-established fluorophores such as CPM that are not compatible with commercial machines. The flexibility of the device is given by the fully automated version with tailored software and a simplified version only controlling the heating process. Our setup is beneficial for method diversity as well as in pricing when compared to the purchasable device making it an attractive alternative for every days use.

## Electronic supplementary material

Supplementary Information
