# Supplementary material for: Low cost, microcontroller based heating device for multi-wavelength differential scanning fluorimetry
Source: Sci Rep. 2018 Jan 23;8:1457. doi: 10.1038/s41598-018-19702-6 (PMC5780519; doi:10.1038/s41598-018-19702-6)
Supplement: Supplementary file 1 — Supplementary Information [file 41598_2018_19702_MOESM1_ESM.doc]

**Supplementary Material for**

**Low cost, multi-wavelength microcontroller based heating device for differential scanning fluorimetry**

by

Jo Hoeser, Emmanuel Gnandt & Thorsten Friedrich

Contents:

**Figure S1**: Rendered view of heater assembly in the LS-55 fluorescence spectrometer (Perkin-Elmer).

**Figure S2**: Interconnection of the components of the microcontroller based setup.

**Table S1**: List of materials including approximate costs.


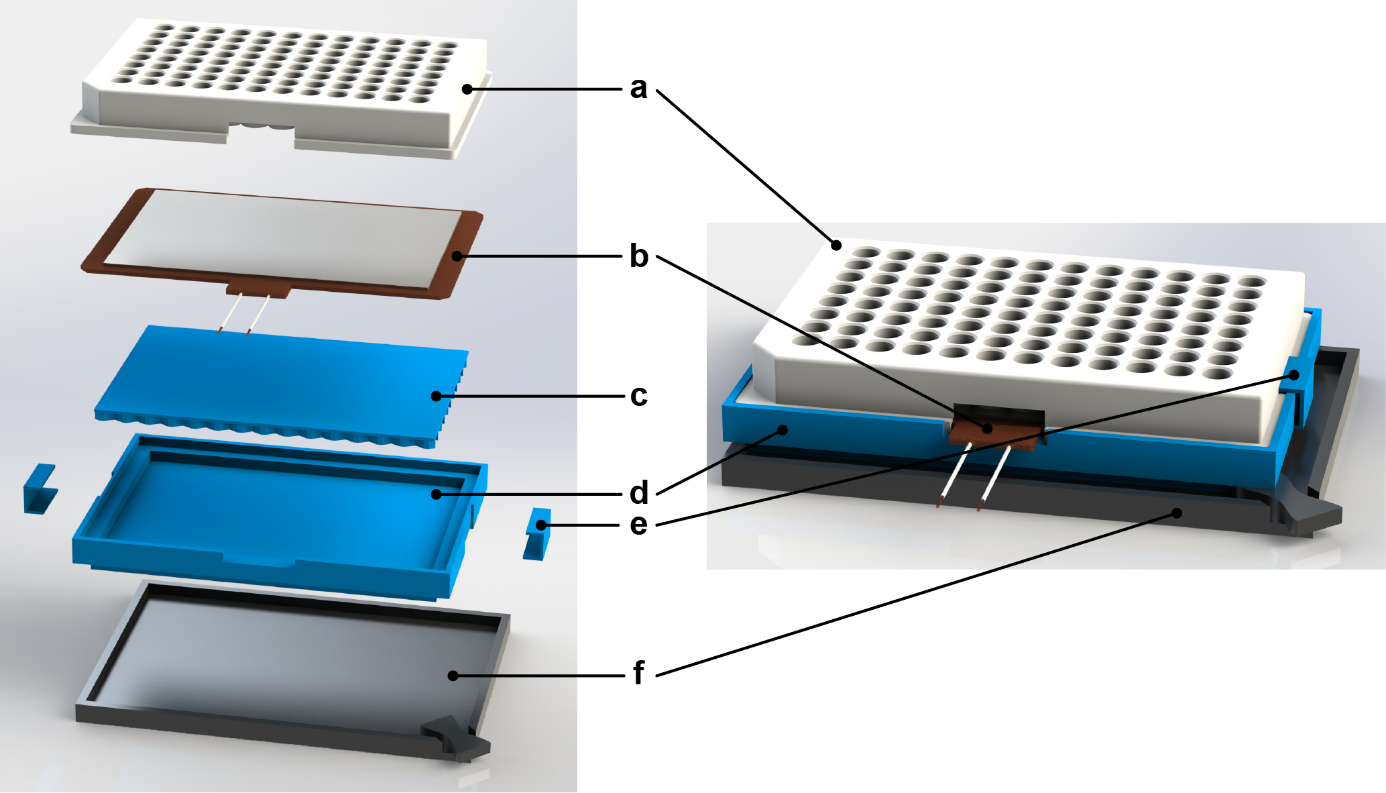


**Figure S1**: Rendered view of heater assembly in the LS-55 fluorescence spectrometer (Perkin-Elmer). All parts displayed in blue were 3D printed using ABS plastic. (a) 96-well plate with groove for heater cables. (b) Silicon heat pad with attached stainless steel sheet. (c) Inlay for plate holder adapter. (d) Adapter piece to thermally insulate heater assembly from plate holder. (e) Clamps to attach heater assembly to adapter piece. (f) Plate holder of the plate reader accessory.


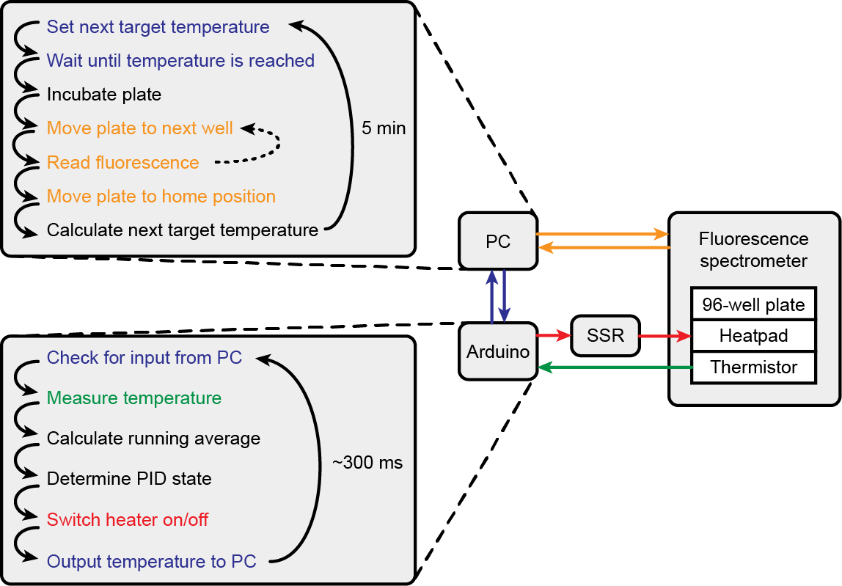


**Figure S2**: Interconnection of the components of the microcontroller based setup. The software orchestrated sequence of individual steps on PC and Arduino is shown by curved arrows. The looped measurement of individual wells is shown by a dashed arrow. Communication between components is shown by straight arrows. SSR: Solid-state relay.

**Table S1**: List of materials including approximate costs.

| **Part** | **Cost (€)** |
| --- | --- |
| Custom made silicon heat pad; 83x125 mm, 12 V, 28 W | ~200 |
| 1mm stainless steel sheet; 100x150 mm | ~1 |
| Generic NTC Thermistor; 100 kΩ, β=3950 | ~1 |
| Generic DC-DC Solid-State Relay; 3-32 V/5-220 V 10 A | ~10 |
| Arduino Uno R3 | ~20 |
| 3D printed parts | ~5 |
| Prototyping board, wires, resistors, solder, Kapton tape, etc | ~10 |
| Total | <250 |
